# Comorbidities and Patient Outcomes in Carbapenem-Resistant Gram-negative Bacterial Infections

**DOI:** 10.1093/ofid/ofag551

**Published:** 2026-09-15

**Authors:** Jean-Francois Jabbour, Yixuan Li, Angelique E Boutzoukas, Blake Hanson, Yohei Doi, Eric Cober, Erica Herc, Lauren Komarow, Jose M Munita, Martin E Stryjewski, Cesar A Arias, David L Paterson, Michael J Satlin, Vance G Fowler, Robert A Bonomo, David van Duin, for the Antibacterial Resistance Leadership Group, Chip Chambers, Scott Evans, Vance Fowler, Toshi Hamasaki, Robin Patel, Heather Cross, Anthony Harris, Melinda Pettigrew, David van Duin, Helen Boucher, Kim Hanson, Yohei Doi, Thomas Holland, Tom Lodise, Sam Shelburne, Ritu Banerjee, Sara Cosgrove, David Paterson, Ebbing Lautenbach, Sarah Doernberg

**Affiliations:** Division of Infectious Diseases, Department of Medicine, Duke University Medical Center, Durham, North Carolina, USA; The Biostatistics Center, George Washington University, Bethesda, Maryland, USA; Department of Pediatrics, Duke University Medical Center, Durham, North Carolina, USA; Duke Clinical Research Institute, Duke University, Durham, North Carolina, USA; Center for Infectious Diseases and Microbial Genomics, UTHealth, McGovern School of Medicine at Houston, Houston, Texas, USA; Center for Innovative Antimicrobial Therapy, Division of Infectious Diseases, Department of Medicine, University of Pittsburgh School of Medicine, Pittsburgh, Pennsylvania, USA; Department of Infectious Diseases, Cleveland Clinic, Cleveland, Ohio, USA; Division of Infectious Diseases, Department of Medicine, Henry Ford Hospital, Detroit, Michigan, USA; The Biostatistics Center, George Washington University, Bethesda, Maryland, USA; Millennium Initiative for Collaborative Research on Bacterial Resistance (MICROB-R) and Instituto de Ciencias e Innovación en Medicina, Facultad de Medicina Clínica Alemana, Universidad del Desarrollo, Santiago, Chile; Division of Infectious Diseases, Department of Medicine, Centro de Educación Médica e Investigaciones Clínicas (CEMIC), Buenos Aires, Argentina; Division of Infectious Diseases, Houston Methodist Hospital and Houston Methodist Research Institute, Houston, Texas, USA; Center for Infectious Diseases Research, Houston Methodist Hospital and Houston Methodist Research Institute, Houston, Texas, USA; Department of Medicine, Weill Cornell Medical College, New York, New York, USA; ADVANCE-ID, Saw Swee Hock School of Public Health, National University of Singapore, Singapore, Singapore; Division of Infectious Diseases, Department of Medicine, Weill Cornell Medicine, New York, New York, USA; Division of Infectious Diseases, Department of Medicine, Duke University Medical Center, Durham, North Carolina, USA; Duke Clinical Research Institute, Duke University, Durham, North Carolina, USA; Department of Medicine, Case Western Reserve University School of Medicine, Cleveland, Ohio, USA; Research Service, Louis Stokes Cleveland Department of Veterans Affairs Medical Center, Case Western Reserve University VA Center for Antimicrobial Resistance and Epidemiology (Case VA CARES), Cleveland, Ohio, USA; Departments of Pharmacology, Molecular Biology and Microbiology, Biochemistry, and Proteomics and Bioinformatics, Case Western Reserve University School of Medicine, Cleveland, Ohio, USA; Department of Medicine, Louis Stokes Cleveland Department of Veterans Affairs Medical Center, Cleveland, Ohio, USA; Division of Infectious Diseases, Department of Medicine, University of North Carolina School of Medicine, Chapel Hill, North Carolina, USA

**Keywords:** antimicrobial resistance, comorbidities, Gram-negative infections

## Abstract

In an international cohort of hospitalized patients included in the Multi-Drug-Resistant Organism Network with carbapenem-resistant Gram-negative infections, increased age-adjusted Charlson Comorbidity Index scores were associated with increased mortality particularly in those with respiratory and less so in those with bloodstream infections. Cirrhosis, diabetes, and peripheral vascular disease were important contributors.

## Teaser

In patients hospitalized with carbapenem-resistant Gram-negative infections, increased age-adjusted Charlson Comorbidity Index scores were associated with increased mortality. Cirrhosis, diabetes, and peripheral vascular disease were important contributors.

Patients over the age of 70 have more comorbidities and healthcare exposures, placing them at increased risk for multidrug-resistant infections [1]. Outcomes after infections with carbapenem-resistant (CR) organisms are affected by host-related risks in older adults due to immunosenescence and immune exhaustion. The Charlson Comorbidity Index (CCI), introduced in 1987 to categorize comorbid conditions affecting 1-year mortality, remains widely used [2]. The CCI comprises 19 weighted comorbidities, and the total score is calculated by summing the individual scores assigned to each condition. Here, we investigated the association between CCI and its components on outcomes in patients with severe infections due to CR Gram-negative bacteria (GNB).

## METHODS

### Study Design, Patient Population, and Inclusion Criteria

The Multi-Drug-Resistant Organism (MDRO) Network (Clinicaltrials.gov: NCT03646227) studies were multinational, prospective, observational cohort studies that enrolled consecutive hospitalized patients with CR organisms. A secondary analysis was conducted in patients with CR Enterobacterales (CRE), *Acinetobacter baumannii* (CRAb), or *Pseudomonas aeruginosa* (CRPa) enrolled between April 2016 and November 2019 into the Consortium on Resistance Against Carbapenems in *Klebsiella* and Other Enterobacterales (CRACKLE-2) [3], the Prospective Observational *Pseudomonas* Study (POP) [4], and the Study Network of *Acinetobacter* as a Carbapenem-Resistant Pathogen (SNAP) [5], respectively. Patients with bloodstream or respiratory infections as previously described were included, and excluded if all comorbidity data were missing (n = 4). We limited our analysis to blood and respiratory infections, as they have the highest attributable mortality [6]. This cohort includes patients enrolled from 49 study sites in 8 countries (USA n = 30, Australia n = 5, Colombia n = 5, Argentina n = 3, Chile n = 2, Singapore n = 2, Lebanon n = 1, and New Zealand n = 1).

### Ethics Statement

The institutional review boards of all participating centers approved the study. The research was conducted with a waiver of consent.

### Clinical Definitions and Outcomes

Carbapenem resistance was defined as resistance to ertapenem, imipenem, and/or meropenem by local laboratory susceptibility for CRE, and as resistance to meropenem for CRPa and CRAb on central laboratory testing. All comorbidity and outcome variables were abstracted from each participating site's electronic health record, entered by trained research personnel into a standardized case report form, and underwent quality and validation checks. Conditions not explicitly documented as present were assumed to be absent. A validated age-adjusted CCI score was calculated [7]. The primary outcome was 30-day all-cause mortality. We calculated an a priori-defined ordinal 4-level desirability of outcome ranking (DOOR) at 30 days after the index culture [3], considering the following unfavorable events: (1) unsuccessful discharge, including hospitalization or readmission within 30 days of index culture; (2) adverse events: *Clostridioides difficile* infection or renal failure; and (3) persistent symptoms or recurrent infection.

### Statistical Analysis

Patients were stratified into 4 groups based on age-adjusted CCI score: 0–2, 3–4, 5–6, and ≥7 **(**Supplementary Figure 1**)**. Discrete stratification was used because the CCI's scoring system is not normally distributed and is ordinal rather than proportionally scaled (ie, equal point increases do not reflect equivalent changes in risk). Associations between CCI and patient characteristics were evaluated by Kruskal–Wallis test for continuous variables and Pearson χ^2^ for categorical variables. Absolute mortality differences comparing the presence and absence of each comorbidity were estimated, and 95% confidence intervals (CIs) were calculated using the Miettinen–Nurminen score method. Cumulative mortality differences were calculated using sequential dichotomizations of the age-adjusted CCI: (1) score ≥1 vs 0; (2) score ≥3 vs 0–2; (3) score ≥5 vs 0–4; and (4) score ≥7 vs 0–6. A DOOR score was calculated, with the best rank being alive with no undesirable events, followed by being alive with 1 event, alive with 2 or 3 events, and the least desirable outcome being deceased. *P*-values <.05 were considered significant. Statistics were performed using SAS software version 9.4 and R version 4.2.1.

## RESULTS

### Patient Demographics

Across the MDRO Network, 1464 patients with bloodstream (802/1464, 55%) or respiratory (662/1464, 45%) infections were included **(**Table 1**)**, with 66% (961/1464) CRE, 14% (203/1464) CRAb, and 20% (300/1464) CRPa infections. Most patients were male (883/1464, 60%) with a median age of 62 years [first quartile (Q1) 49, third quartile (Q3) 72]. Age-adjusted CCI scores were between 0 and 2 for 28.5% (418/1464), 3 and 4 for 25.5% (374/1464), 5 and 6 for 21% (308/1464), and 7 + for 24% (364/1464). Patients with lower scores acquired infections later during hospitalization [median (Q1, Q3) days (CCI 0–2: 14 (2, 31); 3–4: 11 (1, 30); 5–6: 8 (0, 27.5); 7+: 7 (0, 22) (Kruskal–Wallis *P* < .001)], and had longer overall length of stay [median (Q1, Q3) days (CCI 0–2: 38 (17, 68); 3–4: 30 (13, 53); 5–6: 26 (12, 58.5); 7+: 20 (11, 41) (Kruskal–Wallis *P* < .001)].

**Table 1.** Patient Demographics According to 4-Category Age-Adjusted Charlson Comorbidity Index Score

Characteristic	Age-Adjusted Charlson Comorbidity Index Score					
	0–2 (N = 418)	3–4 (N = 374)	5–6 (N = 308)	7+ (N = 364)	Total (N = 1464)	*P*-Value^a^
Sex, male	256 (61%)	237 (63%)	185 (60%)	205 (56%)	883 (60%)	.257
Median age at culture, years (Q1, Q3)	40 (25, 52)	63 (56, 69)	68 (60, 75)	73 (65, 80)	62 (49, 72)	
Hispanic ethnicity	145 (35%)	91 (24%)	65 (21%)	51 (14%)	352 (24%)	<.001
Country or region						<.001
United States of America	269 (64%)	265 (71%)	233 (76%)	312 (86%)	1079 (74%)	
South/Central America	121 (29%)	90 (24%)	55 (18%)	31 (9%)	297 (20%)	
Middle East	16 (4%)	12 (3%)	8 (3%)	11 (3%)	47 (3%)	
Australia/Singapore	12 (3%)	7 (2%)	12 (4%)	10 (3%)	41 (3%)	
Origin of admission						<.001
Home	241 (58%)	217 (58%)	159 (52%)	173 (48%)	790 (54%)	
Transfer from another hospital	120 (29%)	81 (22%)	52 (17%)	66 (18%)	319 (22%)	
Long-term care (acute and chronic)	48 (11%)	70 (19%)	88 (29%)	122 (34%)	328 (22%)	
Admitted to ICU prior to first positive culture	320 (77%)	252 (67%)	208 (68%)	252 (69%)	1032 (71%)	.01
Patient location at first positive culture						.003
Emergency department	26 (6%)	37 (10%)	47 (15%)	46 (13%)	156 (11%)	
ICU	237 (57%)	202 (54%)	160 (52%)	197 (54%)	796 (54%)	
Medical ward	103 (25%)	95 (25%)	80 (26%)	102 (28%)	380 (26%)	
Organisms						.757
CRE	274 (66%)	246 (66%)	201 (65%)	240 (66%)	961 (66%)	
CRPa	92 (22%)	80 (21%)	58 (19%)	70 (19%)	300 (20%)	
CRAb	52 (12%)	48 (13%)	49 (16%)	54 (15%)	203 (14%)	
Source						.796
Blood	229 (55%)	207 (55%)	174 (56%)	192 (53%)	802 (55%)	
Respiratory	189 (45%)	167 (45%)	134 (44%)	172 (47%)	662 (45%)	
Median days from admission to culture (Q1, Q3)	14 (2, 31)	11 (1, 30)	8 (0, 27.5)	7 (0, 22)	10 (1, 28)	<.001
Median days from culture to discharge or death (Q1, Q3)	17 (7, 35)	13 (6, 30)	13 (6, 25.5)	12 (6, 23)	14 (6, 28)	<.001
Median days from admission to discharge or death (Q1, Q3)	38 (17, 68)	30 (13, 53)	26 (12, 58.5)	20 (11, 41)	28 (13, 56.5)	<.001

CRAb, carbapenem-resistant *Acinetobacter baumannii*; CRE, carbapenem-resistant Enterobacterales; CRPa, carbapenem-resistant *Pseudomonas aeruginosa*; ICU, intensive care unit; Q1, first quartile; Q3, third quartile.

Unless otherwise specified, values represent n (%).

^a^ *P*-values were calculated by Kruskal–Wallis test for continuous variables and Pearson χ^2^ for categorical variables.

### CCI Scores and Associated Outcomes

All-cause 30-day mortality was 31% (450/1464) and increasing CCI scores were significantly associated with increases in 30-day mortality rate: 22% (CCI 0–2) to 32% (CCI 3–4), 30% (CCI 5–6), and 40% (CCI 7+) (Mantel–Haenszel χ^2^  *P* < .001) **(**Supplementary Figure 2**)**. Across the cohort, 31% (454/1464) of patients were alive without events at 30 days from the index culture, 19% (284) were alive with 1 event, and 19% (276) were alive with 2 or more events **(**Supplementary Figure 2**)**. The distribution of DOOR levels also varied significantly across CCI scores (Kruskal–Wallis *P* = .003). Patients with lower scores (CCI 0–2 and 3–4) were more likely to be alive without events at 30 days (33% for both) compared to those with higher scores (CCI 5–6: 31%; 7+: 26%) **(**Supplementary Figure 2**)**.

### Mortality Differences Between Sources of Infection and Individual Comorbidities

**Figure 1.**
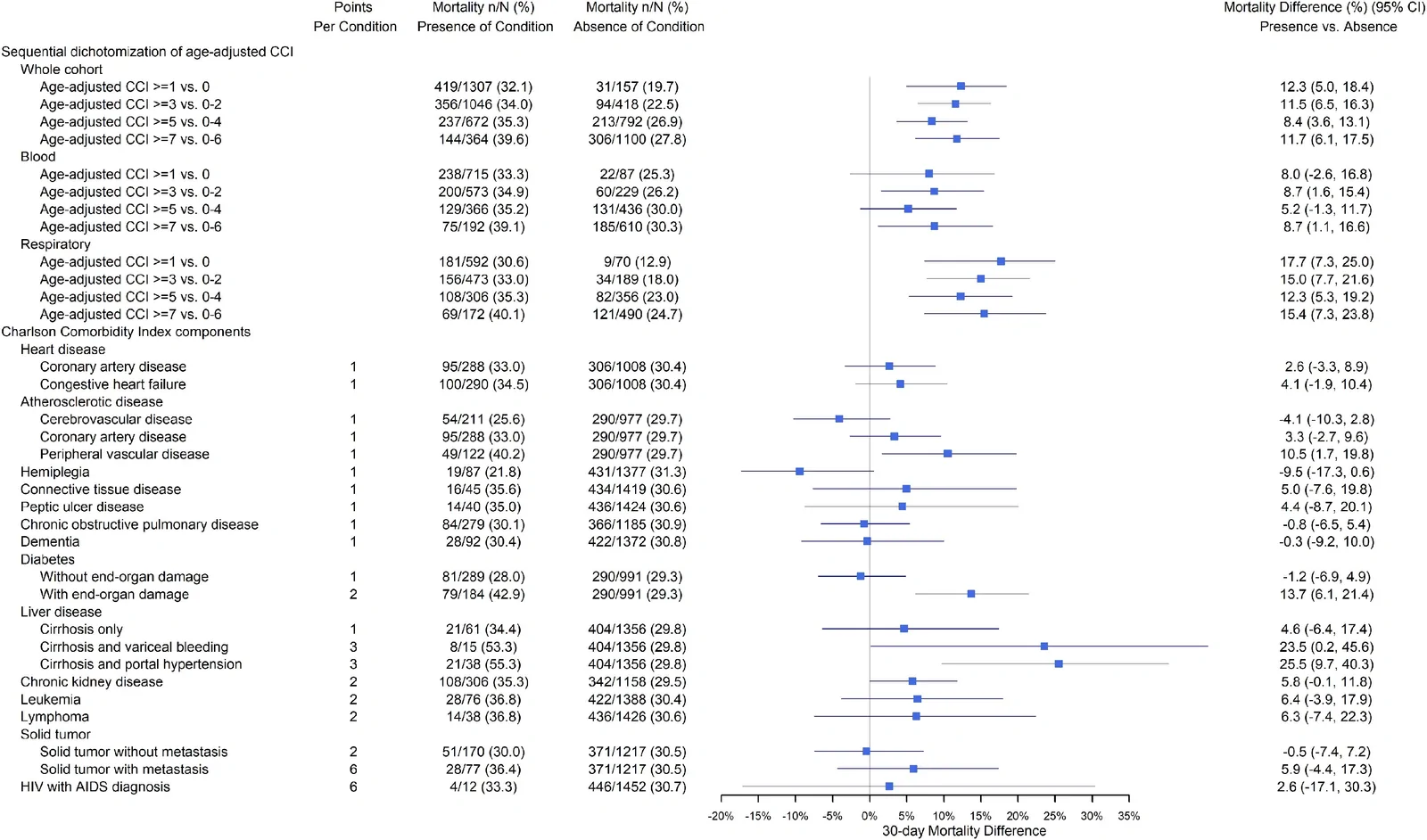
30-day mortality differences and forest plot of Charlson Comorbidity Index score components among blood and respiratory infections. Comorbid conditions and sequential dichotomizations of the age-adjusted CCI are plotted with their corresponding 30-day mortality differences (presence vs absence), with 95% confidence intervals. The highest differences are observed in diabetes with end-organ damage, peripheral vascular disease, and cirrhosis with portal hypertension. Abbreviations: AIDS, acquired immunodeficiency syndrome; CCI, Charlson Comorbidity Index; CI, confidence interval; HIV, human immunodeficiency virus; n/N, number of deaths/total number of patients.

Across the overall cohort, higher CCI scores correlated with greater mortality differences between the presence and absence of comorbidities. When stratified by infection source, significant differences were observed among patients with respiratory infections [17.7% (CCI ≥1 vs 0), 15% (CCI ≥3 vs 0–2), 12.3% (CCI ≥5 vs 0–4), and 15.4% (CCI ≥7 vs 0–6)] (Figure 1). Differences in mortality among patients with bloodstream infections were less pronounced and did not meet significance in 2 strata (Figure 1).

Regarding specific comorbid conditions, patients without diabetes had a 30-day mortality of 29.3% (290/991), compared to 28% (81/289) in diabetics without end-organ damage, and 42.9% (79/184) in diabetics with end-organ damage (*P* < .001) **(**Figure 1**)**. Mortality differences were also seen in patients with or without cirrhosis with portal hypertension (mortality difference 25.5%, 95% CI 9.7%–40.3%; *P* < .01), and peripheral vascular disease (PVD) (mortality difference 10.5%, 95% CI: 1.7%–19.8%; *P* < .001).

## DISCUSSION

In patients with CR GNB infections, the presence of comorbidities was associated with increases in mortality in respiratory infections. This is consistent with prior studies on pneumonia caused by other pathogens. In one study of adults with community-acquired pneumonia, comorbidities that independently increased mortality included alcohol abuse, tumor, cerebrovascular disease, heart failure, dementia, nervous system disease, and renal insufficiency [8]. Similarly, in adults ≥50 years hospitalized with community-acquired pneumonia, mortality rose with increasing number of comorbidities, particularly when high-risk (eg, malignancy or immunodeficiency) and at-risk (eg, cardiac, liver, or respiratory) conditions coexisted [9]. We observed a less pronounced relationship between CCI and mortality in patients with bloodstream infections. Prior studies on bloodstream infections caused by other, generally more antibiotic-susceptible pathogens, suggest that patients with more comorbidities are at increased risk for mortality [10]. Additionally, the CCI was found to be an independent predictor of 14-day clinical failure, including mortality before 14 days from infection, in patients with CR *Klebsiella pneumoniae* bacteremia [11].

Specific comorbidities conferred measurable mortality risks among patients with CR GNB infections. Cirrhosis with portal hypertension, diabetes with end-organ damage, and PVD demonstrated the largest mortality differences. These associations are consistent with prior studies showing nearly 4-fold higher mortality in cirrhotic patients, higher infection-related mortality in diabetics due to impaired immune response, and worse outcomes in patients with PVD after sepsis [12, 13, 14].

In our study, higher CCI scores were also associated with earlier infection onset, possibly due to more healthcare exposure prior to index hospitalization, and with shorter stays, but culture-to-discharge times showed no consistent trends. The CCI is a well-validated and widely used research tool with strong inter-rater reliability [15]. We used age-adjusted CCI because it integrates the compounded effects of aging and comorbidities on mortality [7]. However, as the CCI was developed over 40 years ago, its weighting may not reflect contemporary disease prognoses or treatment advances, and dynamic updates could improve its predictability.

Limitations to our study include potential biases inherent in retrospective data collection. Moreover, the DOOR analysis focuses on 3 undesirable events, but other relevant clinical outcomes, like quality-of-life post-discharge or long-term functional outcomes, were not included. Multivariable analyses adjusting for other covariates were not done due to limited event numbers, which restricts statistical power and increases the risk of model overfitting. In cases where the number of patients is low (eg, patients with HIV/AIDS), our estimates have wide confidence intervals, hindering definitive interpretation. Since this observational study includes only patients with positive cultures, attributable mortality cannot be distinguished by design, and outcomes are not compared to uninfected, comorbidity-matched patients. The strength of this study lies in the large, multicenter network, which allows for generalizability of our findings across patient populations.

Comorbidities have a considerable impact on the outcomes of patients with CR infections, especially those of the respiratory tract. Patients with diabetes mellitus, cirrhosis, PVD, and those with a high cumulative comorbidity burden are particularly vulnerable.

## Supplementary Material

ofag551_Supplementary_Data

## Supplementary Data

Supplementary materials are available at *Open Forum Infectious Diseases* online. Consisting of data provided by the authors to benefit the reader, the posted materials are not copyedited and are the sole responsibility of the authors, so questions or comments should be addressed to the corresponding author.
