# Supplementary material for: Comorbidities and Patient Outcomes in Carbapenem-Resistant Gram-negative Bacterial Infections
Source: Open Forum Infect Dis. 2026 Sep 15;13(9):ofag551. doi: 10.1093/ofid/ofag551 (PMC13576414; doi:10.1093/ofid/ofag551)
Supplement: Supplementary file 1 [file ofag551_supplementary_data.docx]

**Supplementary Material**

# **SUPPLEMENTAL FIGURE 1**

**Title:** Age-adjusted Charlson Comorbidity Index calculation.

**Legend:** The Charlson Comorbidity Index comprises of comorbid condition with assigned weighted scores. The total CCI score is calculated by summing the weights of present conditions. Age-adjustment is performed by adding a point for each decade of age over 40. Patients are then stratified into four groups based on their final score (0–2, 3–4, 5–6, and 7+).

**Abbreviations:** CCI, Charlson Comorbidity Index; CKD, chronic kidney disease.

# **SUPPLEMENTAL FIGURE 2**

**Title:** Desirability of Outcome Ranking (DOOR) at 30 days in 4-category age-adjusted CCI Score and 30-day mortality of the 4-category Charlson comorbidity Index score

**Legend:**

*Left panel:* Distribution of 30-day outcomes by CCI group, categorized as alive without events, alive with 1 event, alive with 2 or 3 events, and dead. Increasing CCI scores were associated with a higher proportion of adverse outcomes and mortality.
*Right panel:* 30-day mortality increases progressively with higher age-adjusted CCI scores, from 22% in the 0–2 group to 40% in the 7+ group.

**Abbreviations:**
CCI, Charlson Comorbidity Index.
